# Insight into Unprecedented Diversity of Cyanopeptides in Eutrophic Ponds Using an MS/MS Networking Approach

**DOI:** 10.3390/toxins12090561

**Published:** 2020-08-31

**Authors:** Andreja Kust, Klára Řeháková, Jaroslav Vrba, Vincent Maicher, Jan Mareš, Pavel Hrouzek, Maria-Cecilia Chiriac, Zdeňka Benedová, Blanka Tesařová, Kumar Saurav

**Affiliations:** 1 Laboratory of Algal Biotechnology-Centre Algatech, Institute of Microbiology of the Czech Academy of Sciences, 37901 Třeboň, Czech Republic; kust@alga.cz (A.K.); mares@alga.cz (J.M.); hrouzek@alga.cz (P.H.); 2 Biology Centre of the Czech Academy of Sciences, Institute of Hydrobiology, 37005 České Budějovice, Czech Republic; klara.rehakova@hbu.cas.cz (K.Ř.); jaroslav.vrba@prf.jcu.cz (J.V.); cecilia.chiriac@icbcluj.ro (M.-C.C.); 3 Institute of Botany of the Czech Academy of Sciences, 37901 Třeboň, Czech Republic; 4 Faculty of Science, University of South Bohemia, 37005 České Budějovice, Czech Republic; 5 Nicholas School of the Environment, Duke University, Durham, NC 27710, USA; vincent.maicher@duke.edu; 6 ENKI, o.p.s. Třeboň, Dukelská 145, 37901 Třeboň, Czech Republic; benedova@enki.cz (Z.B.); blanka.tesarova@prirodou.cz (B.T.); 7 Faculty of Agriculture, University of South Bohemia, Applied Ecology Laboratory, 37005 České Budějovice, Czech Republic

**Keywords:** cyanobacteria, cyanopeptides, harmful bloom, liquid chromatography-tandem mass spectrometry, global natural product social networking (GNPS), dereplication strategy

## Abstract

Man-made shallow fishponds in the Czech Republic have been facing high eutrophication since the 1950s. Anthropogenic eutrophication and feeding of fish have strongly affected the physicochemical properties of water and its aquatic community composition, leading to harmful algal bloom formation. In our current study, we characterized the phytoplankton community across three eutrophic ponds to assess the phytoplankton dynamics during the vegetation season. We microscopically identified and quantified 29 cyanobacterial taxa comprising non-toxigenic and toxigenic species. Further, a detailed cyanopeptides (CNPs) profiling was performed using molecular networking analysis of liquid chromatography-tandem mass spectrometry (LC-MS/MS) data coupled with a dereplication strategy. This MS networking approach, coupled with dereplication, on the online global natural product social networking (GNPS) web platform led us to putatively identify forty CNPs: fourteen anabaenopeptins, ten microcystins, five cyanopeptolins, six microginins, two cyanobactins, a dipeptide radiosumin, a cyclooctapeptide planktocyclin, and epidolastatin 12. We applied the binary logistic regression to estimate the CNPs producers by correlating the GNPS data with the species abundance. The usage of the GNPS web platform proved a valuable approach for the rapid and simultaneous detection of a large number of peptides and rapid risk assessments for harmful blooms.

## 1. Introduction

Cyanobacteria are important primary producers in the food chain with high nutritional value [1] and tend to proliferate, forming dense blooms, scums, and floating mats under favorable environmental conditions [2,3]. Eutrophication and climatic changes have led to increases in the geographical extent, population densities, and duration of cyanobacterial blooms in fresh, brackish, and marine waters [4]. These blooms can be hazardous to humans, animals, and plants due to the production of cyanotoxins apart from disrupting the ecosystem functions, such as nutrient cycles, light availability, dissolved oxygen levels/content, and consequent community reorganization and reduced biodiversity [5]. The most frequently reported cyanotoxins can be classified as cyclic oligopeptides (i.e., microcystins (MCs) and nodularins (NODs)) or alkaloids (i.e., anatoxins and cylindrospermopsin) based on their chemical structures, and as hepatotoxins, neurotoxins, and dermatotoxins based on their mechanism of toxic action in vertebrates [6,7]. The most extensively studied cyanotoxins are cyclic heptapeptides, MCs, produced most often by *Microcystis, Planktothrix,* and *Dolichospermum* (former *Anabaena*) [4]. NODs, cyclic pentapeptides, are structurally related to MCs and are produced mainly by *Nodularia spumigena*. Both MCs and NODs are hepatotoxins, inhibiting serine/threonine protein phosphatases [8]. To date, about forty cyanobacterial genera have been described as potential cyanotoxins producers [9,10], of which the most common bloom-forming genera include *Microcystis, Aphanizomenon, Cylindrospermopsis, Dolichospermum, Nodularia, Planktothrix, Oscillatoria*, and *Trichodesmium* [4,9]. Moreover, more than six hundred peptides or peptidic metabolites (hereafter “cyanopeptides (CNPs)”) have been isolated from cyanobacteria [11], many of which are unknown with regard to their toxic potential and are not regularly monitored during the cyanobacterial bloom events. The co-occurrence of CNPs has been reported during the cyanobacterial proliferation events, and the necessity for extending their regular monitoring has been addressed [12,13,14,15]. CNPs, such as aeruginosins, microginins, cyanopeptolins, anabaenopeptilides, microviridins, anabaenopeptins, and nostophycins, with numerous structural variants are regularly found in cyanobacterial blooms [13,16,17,18]. Recent findings have suggested that metabolomic profiles consisting of different CNPs affect the cohabiting invertebrates and fish populations differently [19,20,21], underlining the need for the expansion of the number of regularly monitored and studied CNPs. 

Early methods for the detection of toxins are based mostly on animal assays using intraperitoneal or intravenous injections on mice [22,23,24]. However, recent advancements in the field of fast and accurate methods, such as high-performance liquid chromatography connected to tandem mass spectrometry with high-resolution mass spectrometry (HPLC-HRMS/MS) and introduction of the global natural product social (GNPS) molecular networking platform, have gained considerable attention towards its application in the field of identification of novel compounds [25]. Further introduction of an in-silico annotation tool (such as Dereplicator+) at the GNPS online workflow has revolutionized the detection of known/unknown natural products by comparing experimental MS/MS spectra against chemical structure databases. These tools enable the analyses and curation of hundreds to thousands of obtained MS/MS data from analytes within the extract, which is almost impossible to analyze manually [26]. Recent application of these tools in the field of annotating metabolites from cyanobacterial bloom has led to the discovery of various novel compounds as well as unknown analogs [27,28,29,30]. 

Hence, the current study was focused on three eutrophic shallow ponds in the South Bohemia region of the Czech Republic and the determination of their phytoplankton composition and metabolomic profiles during the vegetation season. The metabolic composition was determined by leveraging the GNPS online workflow in silico tools and molecular networking to obtain a complete CNP profile of cyanobacterial proliferation of the studied ponds. 

## 2. Results and Discussion

The studied ponds have been in use for fish production since the 16th century. During the 20th century, natural eutrophication and the intensification of fish production increased and led to heavy eutrophication of these water bodies at present [31], resulting in intensified cyanobacterial proliferation during the summer months. We sampled three ponds, KL (Klec), DH (Dehtář), and KV (Kvítkovický), located in South Bohemia, Czech Republic, once per month during the whole vegetation season (six months in total), to investigate their phytoplankton, CNPs composition, and dynamics. The chemical background data of the studied ponds indicated high concentrations of total nitrogen (TN), total phosphorus (TP), and chlorophyll-a (chl-a), illustrating the hypertrophic status of ponds (Table 1) [32]. All three ponds included in the current study showed a high content of chl-a, the primary and dominant photosynthetic pigment used as a proxy for phytoplankton biomass [33], with lowest concentration in KL-Apr (61.0 µg/L) and highest in KL-Jul (376.1 µg/L). Overall, the increase of water temperature and of total nitrogen resulted in higher cyanobacterial proliferation, while chl-a concentrations were correlated with the increase of cyanobacteria and/or diatoms biomass. To study seasonal dynamics of phytoplankton with emphasis on cyanobacterial species composition, we quantified phytoplankton at the species level (wherever clear taxonomical identification was possible) and statistically correlated cyanobacterial taxa with detected CNPs.

### 2.1. Phytoplankton Composition and Seasonal Dynamics

**Figure 1:**
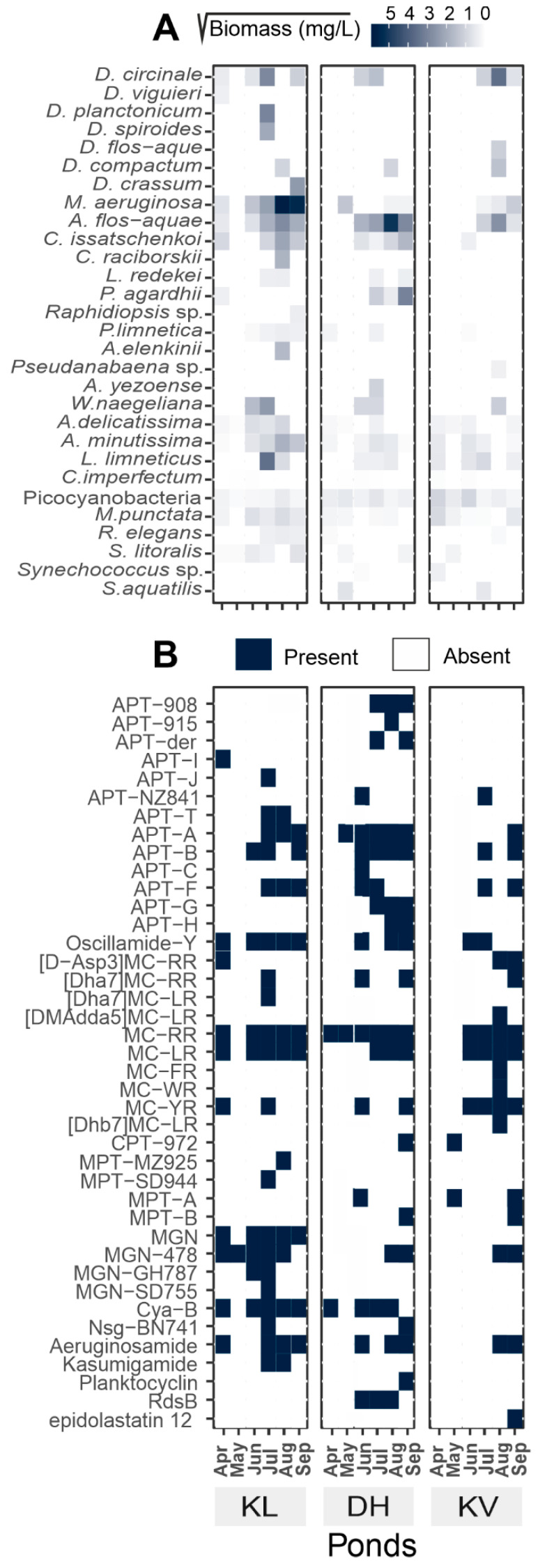
Heat maps showing (**A**) the square root of the biomass in mg/L of different cyanobacterial species in all ponds during all sampled months, and (**B**) the presence/absence of the different cyanopeptides (CNPs) detected in all ponds during all sampled months. The full names of cyanobacterial species can be found in Figure S1.

Phytoplankton of the three studied sites was assigned to classes Chlorophyceae, Cyanophyceae, Cryptophyceae, Bacillariophyceae, Euglenophyceae, Dinophyceae, and Zygnematophyceae (Figure S1A–C). During April and May, phytoplankton of all the three studied sites (KL, DH, KV) was dominated by *Chlorophyceae*, while cyanobacterial biomass did not exceed 3 mg/L of total phytoplankton biomass (Table S1, Figure S1A–C). Total cyanobacterial biomass in KL-Apr was 2.2 mg/L, from which 90.7% was composed of toxigenic taxa *Cuspidothrix issatschenkoi, Microcystis aeruginosa, Dolichospermum circinale* and *viguieri, Aphanizomenon flos-aquae,* and *Planktothrix agardhii* (Figure 1A and Figure S1D). On the other hand, cyanobacterial taxa in DH-Apr and KV-Apr were composed mainly of picocyanobacteria (84.2% and 88.3%, respectively). During May, low cyanobacterial biomass with the dominance of picocyanobacteria was observed in all studied ponds, with the exception of 1.9 mg/L (68.1% of cyanobacterial biomass) of toxigenic *Microcystis aeruginosa* in DH-May. Even though the cyanobacterial biomass was lower during April and May, it still formed an important part of the total phytoplankton biomass in some of the samples, i.e., 17.1%, 6.0%, and 5.9% in KL-Apr, KV-May, and DH-Apr, respectively. The dominance of taxa, which have been reported as CNPs producers, was observed in KL-Jun: *Woronichinia naegeliana* 2.6 mg/L (34.7%) and *Microcystis aeruginosa* 2 mg/L (27.5%); in DH-Jun: *Aphanizomenon flos-aquae* 3.3 mg/L (52.8%) and *Dolichospermum circinale* 1.2 mg/L (19.9%), while KV-Jun was dominated by planktic picocyanobacteria 1.5 mg/L (73.7%) and, in general, had the lowest cyanobacterial biomass (~2 mg/L) in comparison with the other two ponds (Figure S1D,E). 

In July, we detected onset of summer phytoplankton peak (except for DH-Jul), with a record of total phytoplankton biomass of 104.4 mg/L in KL-Jul. KL-Jul exhibited the highest diversity of cyanobacterial taxa without clear dominance of a single cyanobacterial taxon, while the cyanobacteria from DH-Jul (12.6 mg/L) were co-dominated by 5.4 mg/L of *Aphanizomenon floss-aquae*, 2.3 mg/L *Dolichospermum circinale,* and 1.4 mg/L *Planktothrix agardhii.* Unlike the other two ponds, KV-Jul was dominated by Bacillariophyceae with only 3.6 mg/L (9.2%) of total phytoplankton biomass belonging to cyanobacteria, out of which the most abundant were the harmful taxa *Dolichospermum circinale* (1.2 mg/L) and *Aphanizomenon floss-aquae* (1.2 mg/L) (Figure 1A). All three studied sites exhibited the highest cyanobacterial biomass (total phytoplankton was composed of more than 50% cyanobacteria) during August, with the dominance of a single or two toxigenic taxa. While toxigenic cyanobacteria formed a major part of KL-Sep phytoplankton, Bacillariophyceae took over cyanobacteria in DH-Sep and KV-Sept, however, with a still high abundance of toxigenic cyanobacteria (i.e., *Aphanizomenon flos-aquae, Planktothrix agardhii, Microcystis aeruginosa,* and *Dolichospermum circinale).* DH and KV showed similar phytoplankton dynamics to the previous studies with an early spring maximum, followed by phytoplankton depression, with a final summer peak, while KL had its phytoplankton depression in spring months with a summer maximum (Figure S1). Observed phytoplankton development corresponds to previously reported plankton dynamics in shallow eutrophic ponds [34,35].

### 2.2. CNPs Diversity: Molecular Networking 

**Figure 2:**
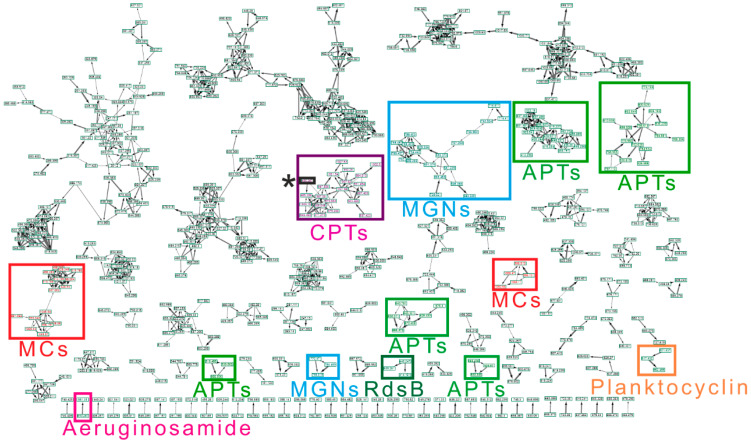
The molecular network generated from HRMS/MS spectra from all the samples of three ponds using global natural product social molecular networking (GNPS) tool. Analytes were compared with the components from the fragmentation pattern library available from the GNPS server. Only clusters of at least two nodes are represented. APTs: anabaenopeptins, MCs: microcystins, CPTs: cyanopeptolins, MGNs: microginins, RdsB: radiosumin_B, *: epidolastatin 12.

Diverse cyanobacterial communities among studied ponds were reflected in the production of a wide array of CNPs. Abundance in CNPs diversity in a given ecosystem could affect any co-existing organisms, especially due to their inhibitory and toxic activities [11,18,36,37]. It has been hypothesized that physiological and ecological relevance of chemotype variability in a single strain, and even higher diversity in natural cyanobacterial bloom population, is advantageous for cyanobacterial dominance against other photoautotrophs and protection against grazing zooplankton [38,39,40]. Applying the online workflow of GNPS for high throughput screening, we detected forty CNPs (Figure 1B and Table S2). A molecular network of 87 clusters was generated using high-resolution mass spectrometry (HRMS) spectra data on the global natural product social molecular networking (GNPS) online workflow (Figure 2). GNPS algorithm automatically aligned and compared each spectrum against the spectra available in the database and then further grouped them by assigning cosine score (0 to 1). 

Further, the obtained molecular network was annotated using an in-silico tool, Dereplicator+. With this tool, it was possible to search all the spectra in the GNPS launched in the molecular network and identify an order of magnitude more natural products than previous dereplication efforts [41]. Eighteen spectrum files were dereplicated, generating 26,220 spectrum scans. A total of 568 peptide-spectrum matches (PSMs) were identified with 321 PSMs, exhibiting a significant score of ≥11.0. The dereplication algorithm enabled us to facilitate natural product discovery by high-throughput peptide natural product identification among large-scale mass spectrometry-based screening platforms [42]. Different analytes were grouped in the same molecular clusters based on the similarity of their fragmentation patterns, with each cluster being potentially specific to the structure of the chemical families. These unidentified ions that belong to annotated clusters are then considered as potential new analogs of their respective molecular family [27]. Additionally, the availability of HRMS/MS spectral data of known cyanotoxins in the GNPS database facilitates the detection process [43,44,45]. This led to the detection of 14 anabaenopeptins (APTs), ten MCs, five cyanopeptolins (CPTs), six microginins (MGNs), two cyanobactins, a dipeptide radiosumin (RdsB), a cyclooctapeptide planktocyclin, and epidolastatin 12 from methanolic extracts of the biomass (Figure 1B, Table S2). Recently, new MC variants have been discovered using an MS-based molecular networking approach from the freshwater cyanobacterial harmful bloom at Green Lake, Seattle [28]. Similarly, numerous reports have been published, where molecular networking is employed to track changes in secondary metabolic profiles, including MCs and other peptides [46,47]. Fragmentation spectra of unknown variants discovered for APTs and MCs using molecular networking have been further manually curated for the identification of diagnostic ion peak [45,48,49,50,51,52].

#### 2.2.1. Anabaenopeptins (APTs)

**Figure 3:**
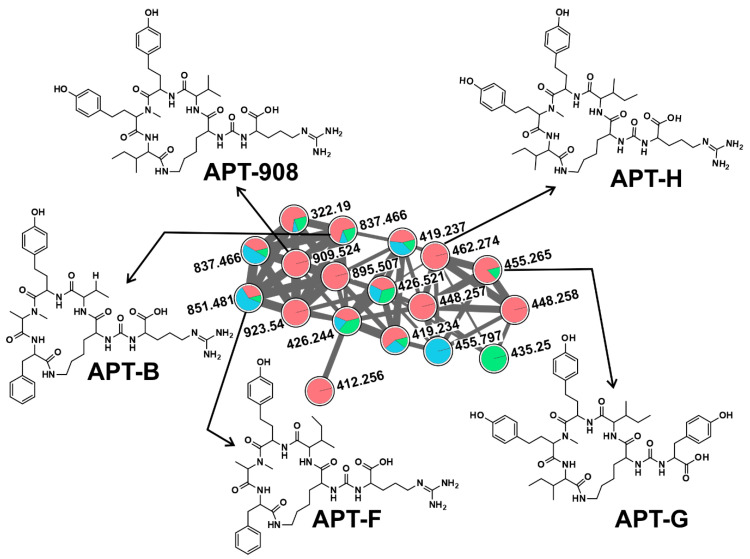
Anabaenopeptin (APT) cluster, formed by the GNPS analysis based on the MS/MS fragmentation spectra obtained from all three sampling sites (Red: DH, blue: KL, green: KV). Here depicted are APTs congener chemical structures detected in this respective cluster, with fragmentation patterns available in the library of the GNPS server. Note that (M + H)^+^ and (M + 2H)^2+^ ions are in the molecular cluster.

Anabaenopeptins are a highly diverse family of cyclic hexapeptides, first described from *Anabaena flos-aquae* NRC 525-17 [53]. They exhibit diverse biological activities; however, studies are mostly focused on their serine protease and chymotrypsin inhibiting activity [54]. They are *N*-methylated and contain a conserved ureido linkage, connecting the side-chain amino acid residue to the D-lys [11]. In the current study, we detected the presence of 14 APTs variants; APT-908, APT-915, APT-I, APT-J, APT-NZ841, APT-T, APT-A, APT-B, APT-C, APT-F, APT-G, APT-H, Oscillamide-Y, and one defined as APT-derivative using a dereplication strategy (Figure 1B, Table S2). Oscillamide-Y (a serine protease inhibitor, isolated from *Planktothrix agardhii* NIES-610; [54]) was detected in ten samples, including KL-Apr with low cyanobacterial abundance (Figure 1). Apt-A and -B were detected nine times, while Apt-F seven times. Apt-A and -B inhibit carboxypeptidase A and protein phosphatase 1 with varying potency, but no inhibition against chymotrypsin, trypsin, and thrombin has been reported [55]. Detected APTs formed five clusters (Figure 2) corresponding to ions, presenting a match with the mass of previously described APTs, suggesting the presence of 34 structural variants, corresponding to potentially new analogs (Figure 2 and Figure 3). Some of the compounds formed single nodes and were removed from the networking. However, we reported here their putative presence based on dereplication. Further, mass spectra of these putative new variants were evaluated for the presence of characteristic ion peak at m/z 84.081 (an immonium fragment ion of lysine) together with other fragment ions of amino acids (Figures S2 and S3) [45,49,50]. The recent increased reports of APTs co-occurrence with MCs raise the attention to this class of CNPs since their impact on the cohabiting aquatic organisms remains unclear, and their ecological role is uncertain [46].

#### 2.2.2. Microcystins (MCs)

Microcystins are cyclic heptapeptides produced, among others, by *Microcystis, Anabaena/Dolichospermum, Nodularia,* and *Oscillatoria*. While the liver is the primary target of MCs, they are also a skin, eye, and throat irritants and immunomodulating agents [56,57]. Microcystin-LR was the first identified cyanotoxin and is the most studied. The WHO has established a provisional guideline value of 1 ug/L for microcystin-LR in drinking water [58]. 

**Figure 4:**
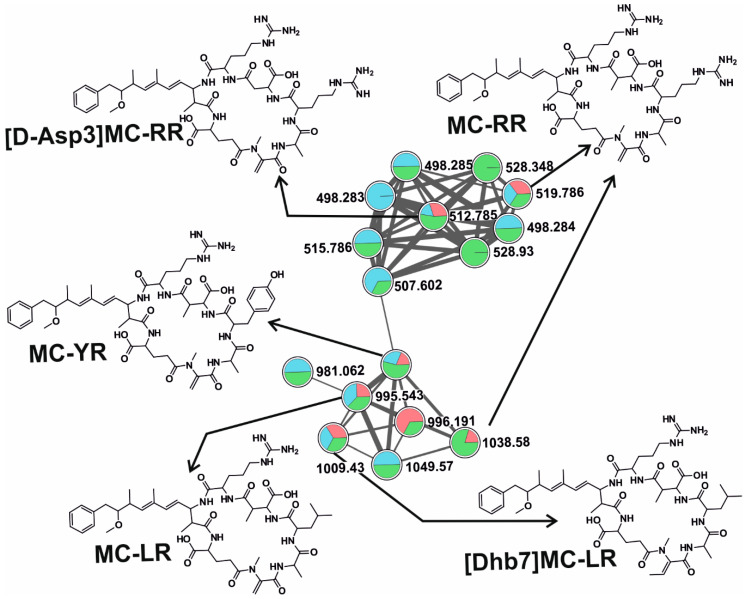
A microcystin (MC) cluster, formed by the GNPS analysis based on the MS/MS fragmentation spectra obtained from all three sampling sites (Red: DH, blue: KL, green: KV). MCs congener chemical structures detected in this respective cluster is depicted here, whose fragmentation patterns are available in the library of the GNPS server. Note that (M + H)^+^ and (M + 2H)^2+^ ions are in the molecular cluster.

We were able to detect putatively ten microcystin congeners—MC-LR, MC-RR, MC-FR, MC-WR, MC-YR, [D-Asp3]MC-RR, [Dha7]MC-RR, [Dha7]MC-LR, [DMAdda5]MC-LR, and [Dhb7]MC-LR (Figure 1B)—using dereplication and molecular networking, forming two clusters (Figure 2). Potentially eleven new analogs were also observed in these clusters, showing distinct but similar fragmentation patterns to those of other known variants spectra present in the GNPS library (Figure 2 and Figure 4). Further, the manual curation of the spectra was performed to identify the most common fragments characterizing MC together with the characteristic ion peak originating from Adda moiety at *m/z* 135.0804 Da, *m/z* 121.0653 for DMAdda, and m/z 163.0759 for ADMAdda (Figure S4 and Figure S5) [48]. MC-RR and MC-LR were detected in all studied ponds, MC-RR was detected in 15 samples, while MC-LR in 12. However, the presence of other congeners was more scattered, with the highest diversity in KV-Aug (eight MC variants).

#### 2.2.3. Cyanopeptolins (CPTs) 

**Figure 5:**
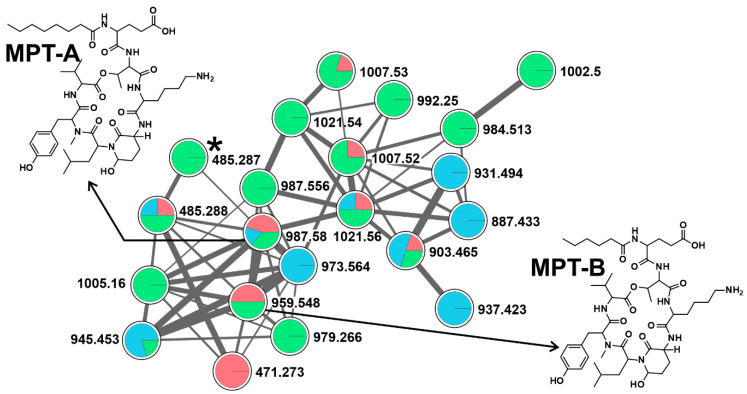
Cyanopeptolins (CPTs) cluster formed by the GNPS analysis based on the MS/MS fragmentation spectra obtained from all three sampling sites (Red: DH, blue: KL, green: KV). CPTs congener chemical structures (MPT: micropeptin) detected in this respective cluster is depicted here together with epidolastatin 12 (*), whose fragmentation patterns are available in the library of the GNPS server. Note that (M + H)^+^ and (M + 2H)^2+^ ions are in the molecular cluster.

CPTs are a diverse class of cyclic depsipeptides previously isolated from *Microcystis* sp. PCC 7806 [59], composed of a six amino acid residue ring structure, a conserved 3-amino-6-hydroxy-2- piperidone (AHP) residue, and a side chain of variable length [59,60]. We detected five CPTs variants (micropeptin (MPT) -MZ925, -SD944, -A, -B, and CPT 972 (Figure 1B) exclusively from summer samples), with a high abundance of cyanobacteria in the phytoplankton community (total nine hits). Two micropeptins and an epidolastatin12 formed a cluster together with compounds that very likely corresponded to potentially eighteen new analogs (Figure 2 and Figure 5). The general activities reported from CPTs are protease inhibitory, fungicidal, cytotoxic, and antitumor activities [61], and the recent elucidation of the molecular basis of AHP-cyclodepsipeptides has opened new possibilities for customizing them as serine protease-specific inhibitors [60]. 

Another peptide, epidolastatin 12, detected in the current study, formed a cluster together with CPTs (Figure 5). Dolastatins were originally reported from the mollusk *Dolabella auricularia* [62]; however, their structural variants were found to be produced by axenic cyanobacteria, implying the possibility that even the first reported dolastatin is produced by cyanobacteria [63,64]. It has been reported as an epimer of dolastatin 12 isolated from marine *Lyngbya majuscula/Schizothrix calcicola* cyanobacterial assemblages [63]. The detection of dolastatins epimer in a single sample (KV-Sep), is to our knowledge, the first report of the dolastatin variant detected from a freshwater source. 

#### 2.2.4. Microginins (MGNs)

**Figure 6:**
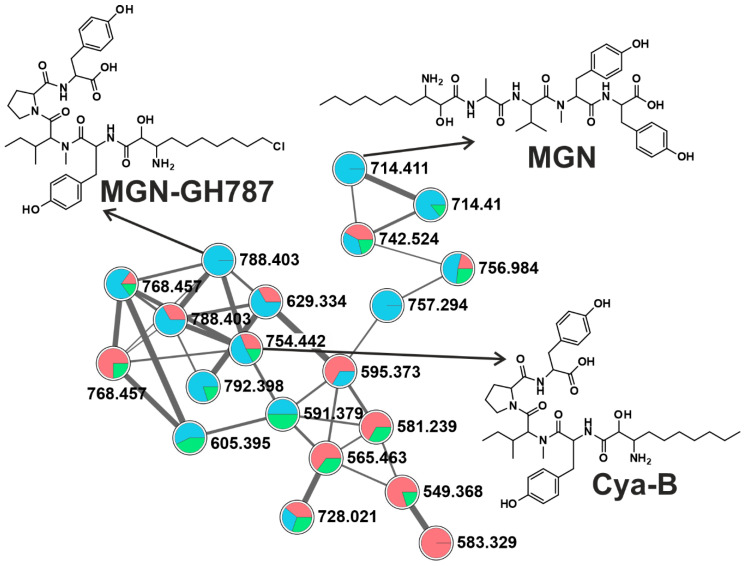
Microginin (MGN) compounds clustered together by the GNPS analysis based on the MS/MS fragmentation spectra obtained from all three sampling sites (Red: DH, blue: KL, green: KV). Selective known MGNs congener chemical structures detected in this respective cluster is depicted here, whose fragmentation patterns are available in the library of the GNPS server. Note that (M + H)^+^ and (M + 2H)^2+^ ions are in the molecular cluster.

Microginins are linear pentapeptides originally isolated from *Microcystic aeruginosa* NIES-100 as an angiotensin-converting enzyme inhibitor [65]. We detected six MGNs variants (MGN, MGN-478, -GH787, -SD755, cyanostatin B (Cya-B), nostoginin BN741 (NSG-BN741)) throughout the sampling season (Figure 1B). In KL-Jul, the most cyanobacterial diverse sampling point (16 taxa, Figure 1), we detected six MGNs variants matching with the library spectra match together with seventeen putative variants forming two clusters (Figure 2 and Figure 6). One cluster comprising three variants is depicted in Figure 6. Similarly, to MCs and APTs, MGNs were detected, also in samples with low cyanobacterial biomass (KL-Apr, -May, DH-Apr). The most frequently detected variants were MGN-478 and Cya-B, both detected in nine samples. The biological activity among MGN variants also varies widely; for example, Cya-B is reported as an aminopeptidase M inhibitor [54], whereas MGN-478 has not exhibited any protease inhibitory activity [66]. 

### 2.3. CNPs Composition and Seasonal Dynamics

Cyanobacterial blooms are formed by diverse coexisting cyanobacterial species, resulting in the production of a wide array of CNPs, altering the natural habitat with their toxic activities [11,18,36,37,67]. Recent studies have reported the co-production of diverse CNPs with MCs [14], suggesting high chemotype variability in a single strain and even higher in natural cyanobacterial blooms [68,69].

The obtained array of CNPs (MCS, APTs, CPTs, MGNs) in our study, in general, corresponds to the expected chemical composition of cyanobacterial blooms dominated by commonly reported toxigenic planktic taxa, such as *Microcystis*, *Dolichospermum*, *Aphanizomenon*, and *Planktothrix* (Table S1) [14,55,70]. However, the amino-protease inhibitor Nsg-BN741 has been previously reported only from periphytic and terrestrial heterocytous cyanobacteria *Nostoc* [71]; thus, it was surprising to find it in the planktic environment (ponds KL and DH). Despite the absence of *Plectonema radiosum* and *Planktothrix rubescens* in the phytoplankton in the DH pond, we detected the presence of RdsB and planktocyclin [72,73].

In the ponds, we detected also two cyanobactins, unlike other CNPs reported here, produced ribosomally [74]. Cyanobactin kasumigamide, a ribosomal tetrapeptide isolated originally from *Microcystis aeruginosa* [66], occurred only in one pond (KL-Jul and -Aug) in sampling points, which showed the highest cyanobacterial diversity. On the other hand, second cyanobactin, aeruginosamide, reported originally from *Microcystis* [75], occurred in all three ponds in a variety of sampling points with different abundances of *Microcystis aeruginosa* co-occurring with diverse cyanotaxa (Figure 1).

As mentioned above, spring samples of all three ponds were dominated by Chlorophyceae with low cyanobacteria abundance. While no CNPs were detected in KV-Apr, we detected nine CNPs in KL-Apr, where the common toxigenic taxa were present, and two CNPs in DH-Apr without the presence of common CNPs producers. Although detection of the CNPs already in samples with low cyanobacterial biomass has been reported previously [76], they are often neglected for detailed monitoring. The detection of diverse CNPs in our samples further addressed a need for detailed monitoring of water bodies, even in samples with low cyanobacterial abundance.

In the following month, all three ponds were dominated mainly by picocyanobacteria, where we found one CNPs in KL-May and two in DH-May. The increase in cyanobacterial abundance late in the sampling season was reflected by an increase in CNPs. An increase in the diversity of common toxigenic cyanobacterial taxa in June resulted in a higher number of detected CNPs (Figure 1). Particularly, KL-Jun and DH-Jun exhibited higher cyanobacterial biomass and diversity of common toxigenic taxa compared with previous samples; thus, in these sampling points, we detected eight and 13 CNPs, respectively. KV-Jun was less abundant in cyanobacteria, resulting in the detection of only four CNPs. 

The highest diversity of the CNPs was detected during the cyanobacterial proliferation in the months of July, August, and September (Figure 1). The most prolific sample in both cyanobacterial and CNP diversity was KL-Jul. This sampling point was characterized by the presence of 16 cyanotaxa and detection of 20 CNPs, referring that the co-occurrence of several toxigenic taxa would result in higher metabolic diversity [14,15]. 

As mentioned above, *Aphanizomenon flos-aquae* was the most abundant species in DH-Jul, where we detected nine CNPs, while KV-Jul was unlike the other ponds dominated by Bacillariophyceae with the lowest cyanobacterial biomass. Accordingly, we detected lower CNP diversity. Generally, the KV pond exhibited lower cyanobacterial and CNP diversity in comparison with the other two studied ponds.

All ponds showed increased cyanobacterial abundance and dominance of a single taxon in August. *Microcystis aeruginosa*, reported diverse CNPs producer [77,78], was the most abundant cyanobacterial taxon in KL-Aug (55%), where we detected 12 diverse CNPs. Thirteen CNPs were detected in DH-Aug dominated by *Aphanizomenon flos-aquae* (90%), while *Dolichospermum* dominated KV-Aug (50%), showing the presence of 12 CNPs, with the highest detected diversity of MCs congeners (eight) among all samples. Both the dominating taxa have been demonstrated as rich secondary metabolite producers [79,80]. KL-Sept had the highest abundance of *Microcystis aeruginosa* (65% of cyanobacterial biomass) with the presence of 11 more taxa, resulting in the detection of nine CNPs. Known for their high CNPs potential, *Aphanizomenon flos-aquae* and *Planktothrix agardhii* [80,81] were the most abundant species in DH-Sep, where 17 CNPs were found. KV-Sep showed a lower diversity of cyanobacteria when compared with the previous sampling point (KV-Aug) and the other two ponds. Dominated with *Microcystis,* KV-Sep exhibited the highest CNPs diversity (13) when compared with previous sampling points of the same pond.

Among all the samples, four samples (DH-May, KL-Aug, KL-Sep, KV-Sep) were dominated mainly by *Microcystis aeruginosa,* where we detected the presence of different variants of CNPs found in other samples not dominated by *Microcystis aeruginosa*; only MPT-MZ925 and epidolastatin 12 were detected exclusively in KL-Aug and KV-Sep, respectively.

**Figure 7:**
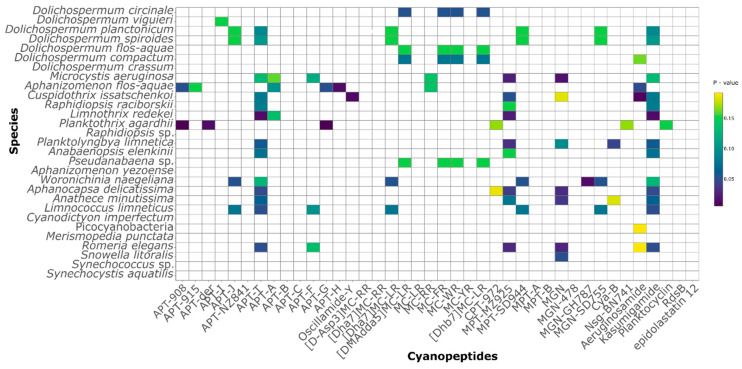
Binary logistic regression of cyanobacterial taxa with distinct CNP production. The full names of CNPs are in Table S2.

Since high cyanobacterial and CNPs diversity co-occurred throughout the sampling campaign, a binary logistic regression was performed in order to correlate specific cyanobacterial taxa with individual CNP occurrence. A number of common toxigenic cyanobacterial taxa exhibited a strong correlation with some of the CNPs; on the other hand, we also observed previously unreported associations (Figure 7). The presence of several reported APTs was significantly correlated with a certain cyanobacterial taxon, previously reported as a producer [55]. APT-915 and APT-H were associated with *Aphanizomenon flos-aquae,* APT-der with *Planktothrix agardhii*, and APT-I with *Dolichospermum viguieri*. Three APTs showed correlation with two taxa, oscillamide Y with *Cuspidothrix issatschenkoi* and *Limnococcus limneticus*, while APT-908 and APT-G were correlated with *Planktothrix agardhii* and *Aphanizomenon flos-aquae*. Other APTs were correlated with three or more taxa, or not correlated to any.

Six out of 10 MCs were correlated with two or more taxa; four MCs (MC-FR, -WR, [DMAdda5]MC-LR, and [Dhb7]MC-LR) detected only in one sample, KV-Aug, showed the same correlation pattern with four cyanobacteria (*Dolichospermum circinale*, *Dolichospermum flos-aquae*, *Dolichospermum compactum*, and *Pseudanabaena* sp.). Similarly, three CPTs showed a correlation with more than one taxon, including not previously reported producers (*Aphanocapsa delicatissima*, *Anathece minutissima*, *Romeria elegans).* MGN-GH787 was correlated with a single taxon, *Woronichinia naegeliana,* previously reported as MGNs producing species [81]. Nsg-BN741, previously reported from *Nostoc*, exhibited a correlation with *Planktothrix agardhii,* while MGN and MGN-SD755 showed a correlation with seven and four taxa, respectively. Furthermore, Cya-B showed association not only with *Planktolyngbya limnetica* but also with *Anathece minutissima* (Figure 7), often reported in cyanobacterial blooms, but never directly associated with CNPs production so far [68,82]. Aeruginosamide was correlated with five cyanobacteria, while planktocyclin, previously reported from *Planktothirx rubescens*, exhibited a correlation with *Planktothrix agardhii*. APT-T and kasumigamide were both detected only in KL-Jul and KL-August and were significantly correlated to the same thirteen cyanotaxa, including non-toxigenic taxa. While *Limnococcus limneticus* (formerly *Chroococcus limneticus*) is generally not considered as toxigenic, it was associated with MGN-SD755, APT-I, -T, -F, [Dha7]MC-LR, Mpt-SD944, MGN-SD755, and kasumigamide. Similarly, picocyanobacterial taxa exhibited a correlation with several CNPs. Picocyanobacteria have been, in general, considered as cyanobacteria with low secondary metabolite potential, although their correlation with MCs production has been repeatedly reported since the eighties [83,84,85].

Jakubowska [82] suggested more in-depth toxicological studies on picocyanobacteria, in general, since CNPs have been already detected in bloom samples with high picocyanobacterial abundance. Only several recent studies have investigated picocyanobacterial’s capability for bioactive secondary metabolites production, such as hepatotoxins, β-N-methylamino-L-alanine (BMAA), lipopolysaccharides, and other bioactive metabolites, i.e. bacteriocins [86]. However, the direct proofs of picocyanobacteria toxicity are still scarce, and authors tend to consider them as non-toxic. 

## 3. Conclusions

In the current study, we implied a non-targeted mass spectrometry approach to determine the metabolome profile of three ponds used for fish farming in the Czech Republic. We detected a range of harmful MCs variants and other potentially harmful CNPs during the entire sampling season and especially (but not exclusively) in samples where cyanobacterial proliferation occurred, which raises concerns on the high presence of harmful CNPs. Usage of the online workflow at GNPS enabled us to identify several classes of CNPs beyond MC. In addition, we were able to putatively determine the presence of several unknown variants of CNPs, which was further evaluated manually by targeting their respective diagnostic ion peak. There is no such study, which investigates/monitor a broad range of CNPs in fish farming ponds, which is regularly used in the fishmarket for human consumption [14]. The current study also aimed to develop this minimal sample treatment method and apply regularly on the freshwater sample monitoring process. Furthermore, detected CNPs (i.e., APT, CPT, MGN) were reported as a co-product along with the MCs; thus, the possible synergistic effect of several compounds produced was addressed. We also introduced a rapid and efficient monitoring approach, combining the GPNS approach and binary logistic regression, for the detection of a wide range of CNPs, even in the samples with low cyanobacterial biomass, which could help to understand the early development and dynamics of CNPs production in aquaculture ponds.

## 4. Material and Methods

### 4.1. Study Sites and Sampling

Three nutrient-rich shallow eutrophic ponds were sampled monthly to cover the growth season, from April until September 2018. The investigated ponds are used for fish production in the Czech Republic: KL (Klec 49.090N, 14.767E, max. depth 2 m, area 0.64 km^2^), DH (Dehtář) 49.006 N, 14.294 E, max. depth 4 m, area 2.28 km^2^), and KV (Kvítkovický 48.963N, 14.337E max. depth 3 m, area 0.24 km^2^). During each sampling point, temperature, pH, conductivity, and transparency were measured (Table 1). Water samples for plankton and background physicochemical analysis were collected, as described previously [32]. Briefly, horizontally integrated mixed water samples from surface water were collected from seven different points with van Dorn sampler (length of 1 m, 6.4 L volume). Chlorophyll a (Chl a) was determined spectrophotometrically after the extraction of samples collected on GF/C filters (Merck KGaA, Darmstadt, Germany), as described elsewhere [87] (Table 1). A subsample (3–5 L) was taken for chemical analysis, and 100 mL was preserved with Lugol’s solution for the analysis of phytoplankton. For the CNPs’ analysis, surface water samples were repeatedly collected with the plankton net (20 µm mesh) until obtaining dense biomass, refrigerated on the way, transferred to the lab, and kept at −80 °C until the analysis.

### 4.2. Phytoplankton Analysis

Biomass of individual phytoplankton taxa was determined in Lugol preserved samples using Utermöhl’s sedimentation method [88] and the inverted microscope (Olympus IMT2, Hamburg, Germany). The abundance of each taxon was multiplied by their respective biovolume calculated from mean cell dimensions using an approximation to geometrical solids [89]. For the taxonomic determination of cyanobacteria, the taxonomic keys by Komárek and Anagnostidis were used [90,91,92].

### 4.3. Crude Extracts Preparation and HPLC-MS/MS Analysis

Crude extracts were prepared following the pre-established protocol [93]. Briefly, freeze-dried biomass of collected pond samples (~20 mg) was ground (with the sea sand) and extracted three times with 75% MeOH in water, followed by bath sonication. Extracts were evaporated under vacuum using a rotary vacuum evaporator (Heidolph, Schwabach, Germany) and dissolved with DMSO to get a final concentration of 4 mg/mL prior to analysis. Thermo Scientific DionexUltiMate 3000 UHPLC (Thermo Fischer Scientific, Waltham, MA, USA) equipped with a diode array detector (DAD) and high-resolution mass spectrometry with electrospray ionization source (ESI-HRMS; Impact HD Mass Spectrometer, Bruker Billerica, MA, USA) was used for the analysis of the crude extracts. HPLC separation was performed on reversed-phase Kinetex Phenomenex C_18_ column (150 × 4.6 mm, 2.6 µm; Phenomenex, Aschaffenburg, Germany) with H_2_O/acetonitrile containing 0.1% HCOOH as a mobile phase. The flow rate during the analysis was 0.6 mL/min. The gradient was as follows: H_2_O/MeOH 85/15 (0 min), 85/15 (in 1 min), 0/100 (in 20 min), 0/100 (in 25 min), and 85/15 (in 30 min). The mass spectrometer settings were as follows: dry temperature 200 °C; drying gas flow 12 L/min; nebulizer 3 bar; capillary voltage 4500 V; endplate offset 500 V. The spectra were collected in the range 20–2000 *m/z,* with the spectra rate 4 Hz. A ramp was set with collision-induced dissociation from 20 to 60 eV on successive *m/z* 200–1200. Data were collected by an initial precursor ion survey scan, followed by product ion generation from precursor ions selected in small isolation windows (≈4 Da wide). Calibration was performed using LockMass 622 (abcr GmbH, Karlsruhe, Germany) as an internal calibration solution and CH_3_COONa clusters at the beginning of each analysis.

### 4.4. Molecular Networking 

The raw data files obtained from HPLC-HRMS/MS analysis were converted to mzXML format using MSConvert from the ProteoWizard suite [94]. A molecular network was created using the online workflow on the GNPS website [26]. The data were filtered by removing all MS/MS fragment ions within +/− 17 Da of the precursor m/z. MS/MS spectra were window filtered by choosing only the top 6 fragment ions in the +/− 50Da window throughout the spectrum. The precursor ion mass tolerance was set to 2 Da and an MS/MS fragment ion tolerance of 0.1 Da. A network was then created where edges were filtered to have a cosine score above 0.65 and more than 6 matched peaks. Further, edges between the two nodes were kept in the network if and only if each of the nodes appeared in each other’s respective top 10 most similar nodes. Finally, the maximum size of a molecular family was set to 100, and the lowest-scoring edges were removed from molecular families until the molecular family size was below this threshold. The spectra in the network were then searched against GNPS spectral libraries. The library spectra were filtered in the same manner as the input data. All matches, kept between network spectra and library spectra, were required to have a score above 0.65 and at least 4 matched peaks. Further, the network was annotated using dereplicator+ to putatively identify the structural details of the compounds present. For annotation using dereplication+, precursor ion mass tolerance of 0.1 Da, fragment ion mass tolerance of 0.01 Da, max charge of 2, min score to consider a PSM as 8.25, and fragmentation mode applied as general_6_1_6.

### 4.5. Statistical Analysis

Statistical analyses were performed in R v. 3.6.1 [95]. The association of specific cyanobacterial species abundance (continuous variable) with distinct cyanotoxin presence/absence (nominal variable) was evaluated using a binary logistic regression in R (‘*glm*’ function from ‘stats’ package) [96]. An asymptotic chi-square statistic based on the deviance was used to assess the goodness-of-fit of each model. *p*-values were adjusted to reduce the number of false positives using the Benjamini–Hochberg procedure [97], with a false discovery rate (FDR) threshold of 0.2. Heatmaps generated using the ‘heatmaply’ function in R (‘heatmaply’ package). The R code developed for the entire analysis is available in Supplementary material as Code 1.

### 4.6. Data Deposition

The mass spectrometry data was deposited in MassIVE public repository (MSV000085840). The molecular networking job can be publicly accessed with the task ID: task=c2034223333641b3a06a72b40d27b2e4.

## Figures and Tables

**Table 1:** Physicochemical characteristics of water of investigated lakes during sampling season. Sampling dates, water temperature, pH, conductivity, transparency, dissolved organic carbon (DOC), total nitrogen (TN), total phosphorus (TP), dissolved organic phosphorus (DRP), and chlorophyll-a (Chl-a) during each sampling. KL stands for Klec, DH for Dehtář, and KV for Kvítkovický.

Locality	Sampling Date	Temperature	pH	Conductivity	Secchi Depth	DOC	TN _#200_	TP _#200_	DRP	Chl-a
		(°C)		(µS/cm)	(cm)	(mg/L)	(mg/L)	(mg/L)	(mg/L)	(µg/L)
KL	24 April 2018	19.3		214	50	12.5	1.84	0.18	0.001	61.0
KL	15 May 2018	19.3	6.9	229	90	16.1	1.57	0.18	0.001	67.3
KL	19 June 2018	21.0	8.5	216	40	14.4	2.34	0.13	0.011	106.1
KL	17 July 2018	21.7	9.4	204	20	16.7	4.37	0.31	0.011	376.1
KL	14 August 2018	23.7	8.9	201	25	19.7	6.61	0.37	0.015	270.2
KL	11 September 2018	18.7	9.4	214	20	20.7	7.77	0.39	0.021	351.7
DH	26 April 2018	18.0		330	50	17.8	1.79	0.22	0.001	65.0
DH	17 May 2018	18.9	8.6	338	40	19.6	2.03	0.24	0.031	68.0
DH	21 June 2018	22.7	8.9	337	65	18.9	1.86	0.20	0.009	71.2
DH	19 July 2018	22.1	8.7	338	40	20.0	2.50	0.30	0.026	104.0
DH	16 August 2018	23.5	9.2	329	35	22.3	3.44	0.29	0.013	161.5
DH	13 September 2018	21.0	9.6	323	40	22.0	4.35	0.15	0.014	114.4
KV	26 April 2018	18.8		313	30	15.6	2.22	0.36	0.094	128.0
KV	17 May 2018	17.3	7.7	372	30	19.4	2.63	0.61	0.422	94.3
KV	21 June 2018	21.8	8.3	349	35	19.3	2.03	0.32	0.060	89.0
KV	19 July 2018	20.9	9.0	334	20	17.7	3.45	0.41	0.022	327.7
KV	16 August 2018	22.1	8.6	347	15	22.7	4.20	0.26	0.041	254.7
KV	13 September 2018	19.7	8.6	341	25	22.0	4.87	0.20	0.014	152.4

## Supplementary Materials

The following are available online at https://www.mdpi.com/2072-6651/12/9/561/s1, Figure S1: Phytoplankton composition of studied ponds throughout the sampling season, Table S1: Biomass of cyanobacterial species and phytoplankton classes in all three studied ponds during the sampling season, Table S2: Detected cyanopeptides (CNPs) in all three studied ponds during the sampling season, Figure S2: HR-MS/MS product ion spectra APT-B in comparison with two unknown variants, highlighting the presence of diagnostic ion peak at m/z 84.0810 (Lys immonium ion) together with other fragment ions of amino acids, Figure S3: HR-MS/MS product ion spectra of protonated known/unknown APTs, forming five clusters (Figure 2), Figure S4: HR-MS/MS product ion spectra MC-RR in comparison with two unknown variants, highlighting the presence of diagnostic ion peak originating from Adda moiety at m/z 135.0804 Da, Figure S5: HR-MS/MS product ion spectra of protonated known/unknown MCs, forming two clusters (Figure 2), Code 1: The R code developed for the entire analysis.
