# Supplementary material for: Insight into Unprecedented Diversity of Cyanopeptides in Eutrophic Ponds Using an MS/MS Networking Approach
Source: Toxins (Basel). 2020 Aug 31;12(9):561. doi: 10.3390/toxins12090561 (PMC7551678; doi:10.3390/toxins12090561)
Supplement: Supplementary file 1 [file toxins-12-00561-s001.pdf]

Supplementary Materials: Insight into Unprecedented Diversity of Cyanopeptides in Eutrophic Ponds Using an MS/MS Networking Approach

Andreja Kust, Klára Řeháková, Jaroslav Vrba, Vincent Maicher, Jan Mareš, Pavel Hrouzek, Maria-Cecilia Chiriac, Zdeňka Benedová, Blanka Tesařová and Kumar Saurav

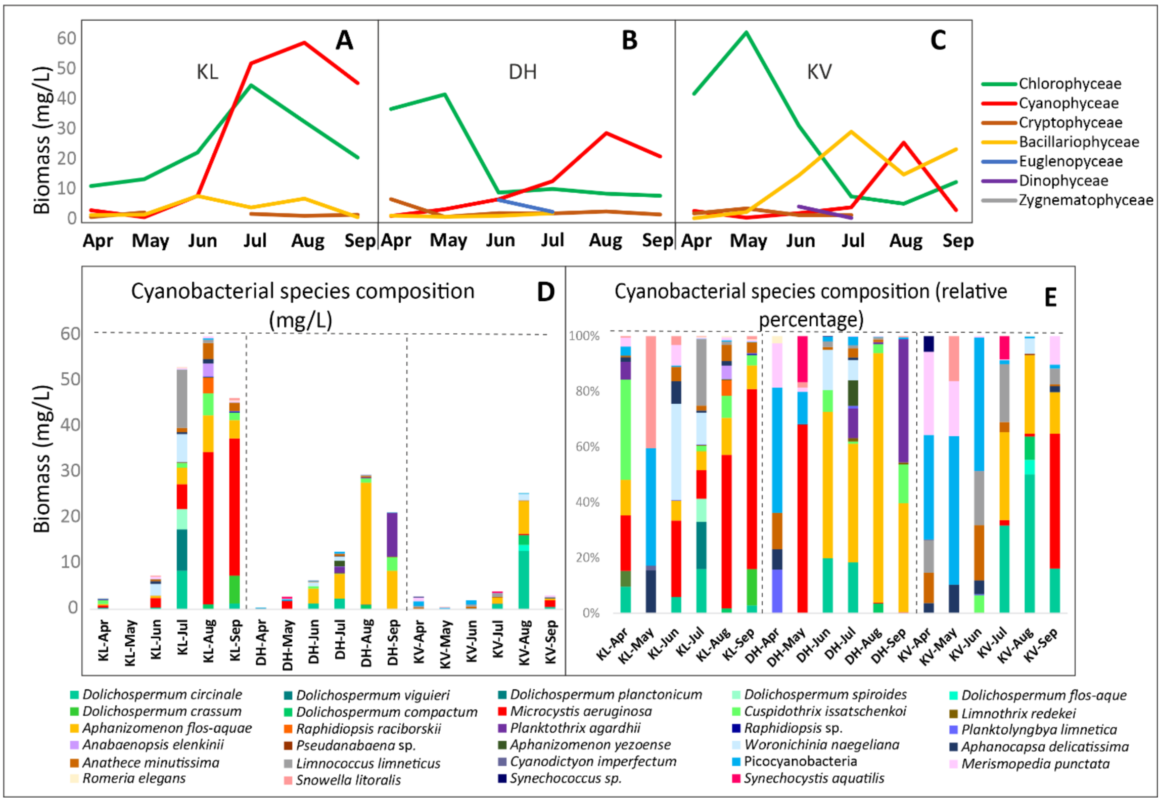

Figure S1. Phytoplankton composition of studied ponds throughout the sampling season. Biomass of phytoplankton among different sampling location **A**) KL **B)** DH, **C)** KV; **D**) biomass of cyanobacterial species; **E**) cyanobacterial relative species composition; KL: Klec, DH: Dehtář, KV: Kvítkovický.

Table S1. Biomass of cyanobacterial species and phytoplankton classes in all three studied ponds during the sampling season. KL: Klec, DH: Dehtář, KV: Kvítkovický.

**Cyanobacteria**	**KL**						**DH**						**KV**					
	**Apr**	**May**	**Jun**	**Jul**	**Aug**	**Sep**	**Apr**	**May**	**Jun**	**Jul**	**Aug**	**Sep**	**Apr**	**May**	**Jun**	**Jul**	**Aug**	**Sep**
Dolichospermum circinale	0.23		0.44	8.48		1.32			1.25	2.32						1.23	12.79	0.49
Dolichospermum viguieri	0.14																	
Dolichospermum planctonicum				9.05														
Dolichospermum spiroides				4.46														
Dolichospermum flos-aquae																	1.35	
Dolichospermum compactum					1.08						1.04						2.14	
Dolichospermum crassum						6.07												
Microcystis aeruginosa	0.49		2.04	5.45	33.40	30.12		1.90			0.10	0.10				0.08	0.28	1.49
Aphanizomenon flos-aquae	0.31		0.53	3.65	8.06	3.96			3.32	5.42	26.63	8.37				1.24	7.23	0.46
Cuspidothrix issatschenkoi	0.88			1.00	4.82	1.70			0.50	0.10	0.95	2.98			0.13			
Raphidiopsis raciborskii					3.38													
Limnothrix redekei				0.17	0.19					0.15		0.16						
Planktothrix agardhii	0.15									1.36	0.24	9.49						
Raphidiopsis sp.						0.19												
Planktolyngbya limnetica			0.02	0.10	0.18	0.13	0.07			0.12					0.01			
Anabaenopsis elenkinii					2.81													
Pseudanabaena sp.																	0.11	
Aphanizomenon yezoense										1.17								
Woronichinia naegeliana			2.57	6.09					0.91	0.91							1.43	
Aphanocapsa delicatissima	0.04	0.01	0.61	0.42	0.96		0.03	0.01		0.12			0.10	0.05	0.10			0.06
Anathece minutissima	0.01		0.38	0.94	3.55	1.84	0.06		0.06	0.42	0.27	0.01	0.30		0.40	0.14		0.02
Limnococcus limneticus			0.00	12.77	0.70				0.13	0.14	0.26		0.32		0.39	0.81		0.17
Cyanodictyon imperfectum	0.003	0.001	0.002					0.001	0.001	0.001			0.006		0.004			
Picocyanobacteria	0.08	0.01	0.03	0.05	0.26	0.04	0.20	0.32	0.11	0.39	0.05	0.14	1.04	0.26	0.97	0.06	0.17	0.04
Merismopedia punctata	0.08		0.55	0.25	0.69	0.31	0.07	0.04		0.03	0.04		0.83	0.10	0.01	0.01		0.31
Romeria elegans				0.14	0.22	0.18	0.01										0.02	
Snowella litoralis	0.02	0.01	0.24	0.11		0.48		0.05				0.08		0.08				
Synechococcus sp.									0.01				0.16					
Synechocystis aquatilis								0.46								0.33		
**Total cyanobacterial biomass**	2.43	0.03	7.40	53.11	60.30	46.33	0.43	2.79	6.28	12.65	29.57	21.33	2.77	0.49	2.01	3.90	25.51	3.06
**Phytoplankton classes**																		

Chlorophyceae	10.80	13.18	22.37	45.59	32.99	20.62	38.00	43.15	8.69	9.91	8.26	7.56	41.81	62.25	31.14	7.58	5.14	12.32
Cyanophyceae	2.43	0.03	7.40	53.11	60.30	46.33	0.43	2.79	6.28	12.65	29.57	21.33	2.77	0.49	2.01	3.90	25.51	3.06
Cryptophyceae	0.10	1.70		1.17	0.51	0.87	6.34	0.13	1.43	1.33	2.05	0.99	1.88	3.56	1.34	1.39		1.16
Bacillariophyceae	0.80	0.92	7.37	3.40	6.47	0.04	0.60	0.16	0.43	1.40		28.71	0.30	2.34	14.59	29.14	14.91	23.27
Euglenopyceae	0.06		1.69				2.01		6.06	1.90		2.47			0.38			0.26
Dinophyceae				1.14								0.32			4.21	0.40		4.38
Zygnematophyceae	0.44		0.38										2.29					
**Total phytoplankton biomass**	14.18	15.83	38.83	104.41	100.27	67.87	47.38	46.24	22.90	27.18	39.88	61.37	46.76	68.64	53.66	42.40	45.57	44.44
% cyanobacteria	17.10	0.22	19.07	50.87	60.13	68.27	0.91	6.04	27.44	46.52	74.15	34.76	5.92	0.72	3.75	9.19	55.99	6.88

Table S2. Detected cyanopeptides (CNPs) in all three studied ponds during the sampling season. KL: Klec, DH: Dehtář, KV: Kvítkovický.

**CNPs**	**KL**						**DH**						**KV**							**Observed Mass**	**Adduct**	**Error,**
	**Apr**	**May**	**Jun**	**Jul**	**Aug**	**Sep**	**Apr**	**May**	**Jun**	**Jul**	**Aug**	**Sep**	**Apr**	**May**	**Jun**	**Jul**	**Aug**	**Sep**	**Total**	(m/z)		**ppm**
APT-908										■	■	■							3	909.5190	M + H	0.2
APT-915											■								1	916.4857	M + H	−4.6
APT-der*										■		■							2	930.4988	M + H	
APT-I	■																		1	760.4628	M + H	−3.2
APT-J				■															1	794.4460	M + H	−1.5
APT-NZ841									■							■			2	842.4466	M + H	−2.3
APT-T				■	■														2	866.5049	M + H	−2.5
APT-A				■	■	■		■	■	■	■	■						■	9	844.4252	M + H	−1.5
APT-B			■	■		■			■	■	■	■				■		■	9	837.4621	M + H	−1.4
APT-C									■										1	809.4555	M + H	0.1
APT-F				■	■	■			■	■						■		■	7	851.4791	M + H	−2.0
APT-G*										■	■	■							3	455.2625	M + 2H	
APT-H											■	■							2	462.2727	M + 2H	−3.5
Oscillamide-Y	■		■	■	■	■			■		■	■			■	■			10	858.4385	M + H	1.3
[D-Asp3]MC-RR	■																■	■	3	512.7967	M + 2H	−2.4
[Dha7]MC-RR				■					■			■						■	4	512.7813	M + 2H	2.1
[Dha7]MC-LR				■															1	981.4770	M + H	−1.6
[DMAdda5]MC-LR*																	■		1	981.5156	M + H	
MC-LR	■		■	■	■	■				■	■	■			■	■	■	■	12	995.5557	M + H	0.3
MC-RR	■		■	■	■	■	■	■	■	■	■	■			■	■	■	■	15	1038.5754	M + H	−2.2
MC-FR																	■		1	1029.5437	M + H	−3.2

MC-WR																	■		1	1068.5547	M+H	−3.1
MC-YR	■			■					■			■			■	■	■	■	8	1045.4971	M+H	−1.7
[Dhb7]MC-LR																	■		1	1009.5744	M+H	−2.7
CPT-972												■					■		2	973.5375	M+H	−2.3
MPT-MZ925					■														1	926.4314	M+H	−2.8
MPT-SD944				■															1	945.5284	M+H	0.8
MPT-A									■								■	■	3	987.5760	M+H	0.1
MPT-B												■						■	2	959.5446	M+H	0.2
MGN	■		■	■	■	■													5	714.4080	M+H	−1.1
MGN-478	■	■	■	■	■						■	■					■	■	9	770.4713	M+H	−1.8
MGN-GH787			■	■															2	788.4005	M+H	−5.1
MGN-SD755				■															1	756.4550	M+H	−1.1
Cya-B	■		■	■	■	■	■		■	■	■								9	754.4390	M+H	−0.6
Nsg-BN741				■								■							2	742.4386	M+H	−0.5
Aeruginosamide	■			■	■	■			■		■	■					■	■	9	561.3475	M+H	−1.1
Kasumigamide				■	■														2	787.3772	M+H	0.2
Planktocyclin												■							1	801.4337	M+H	−1.2
RdsB									■	■	■								3	445.2446	M+H	−0.1
epidolastatin 12*																		■	1	485.2855	M+2H	
**Total CNPs per month**	10	1	8	20	12	9	2	2	13	10	13	17	0	0	4	7	12	13	99			
**Total CNPs per pond**						60						57						36				

Note: Detection of cyanopeptides are presented in the grey fields with their respective month of occurrence, while the white fields represents their absence. APTs: anabaenopeptins, MCs: microcystins, CPTs: cyanopeptolins, MPTs: micropeptins, MGNs: microginins, Cya-B: cyanostatin B, Nsg: nostoginin, and RdsB: radiosumin_B; * Derivatives with poor MS/MS spectra to calculate the error.

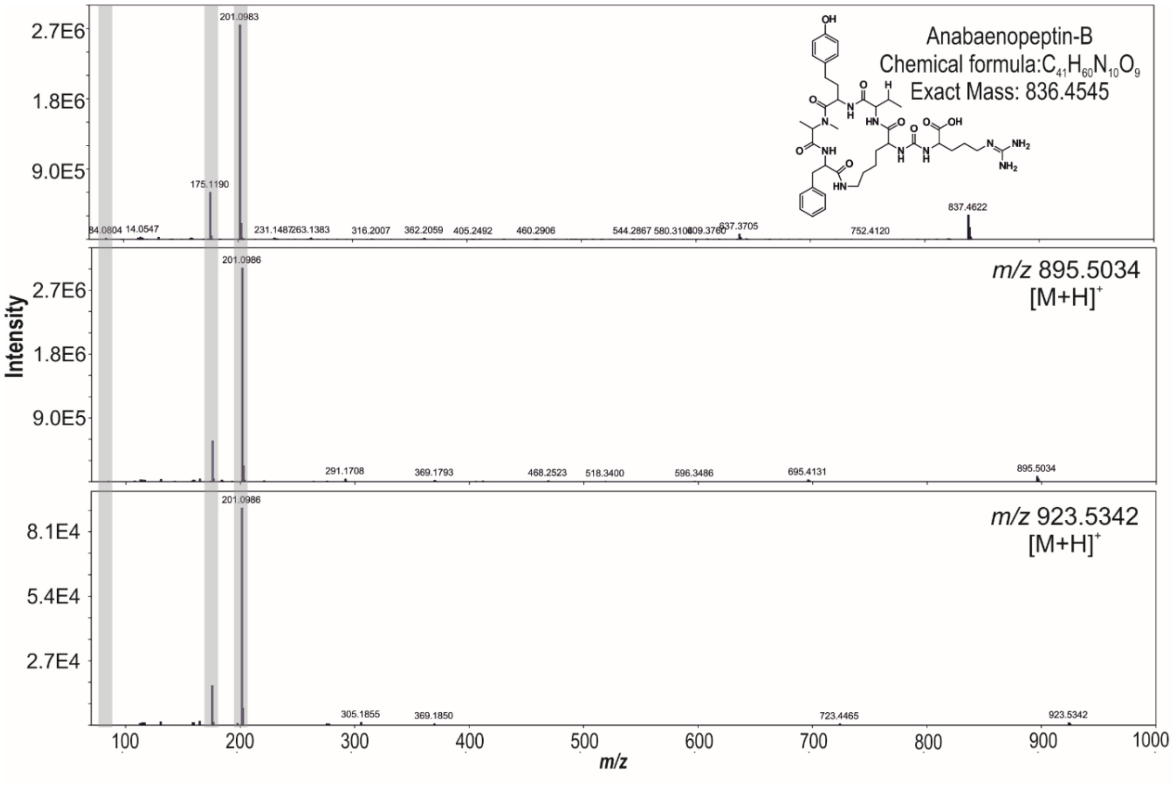

Figure S2. HR-MS/MS product ion spectra APT-B with comparison with two unknown variants highlighting the presence of diagnostic ion peak at m/z 84.0810 (Lys immonium ion) together with other fragment ions of amino acids.

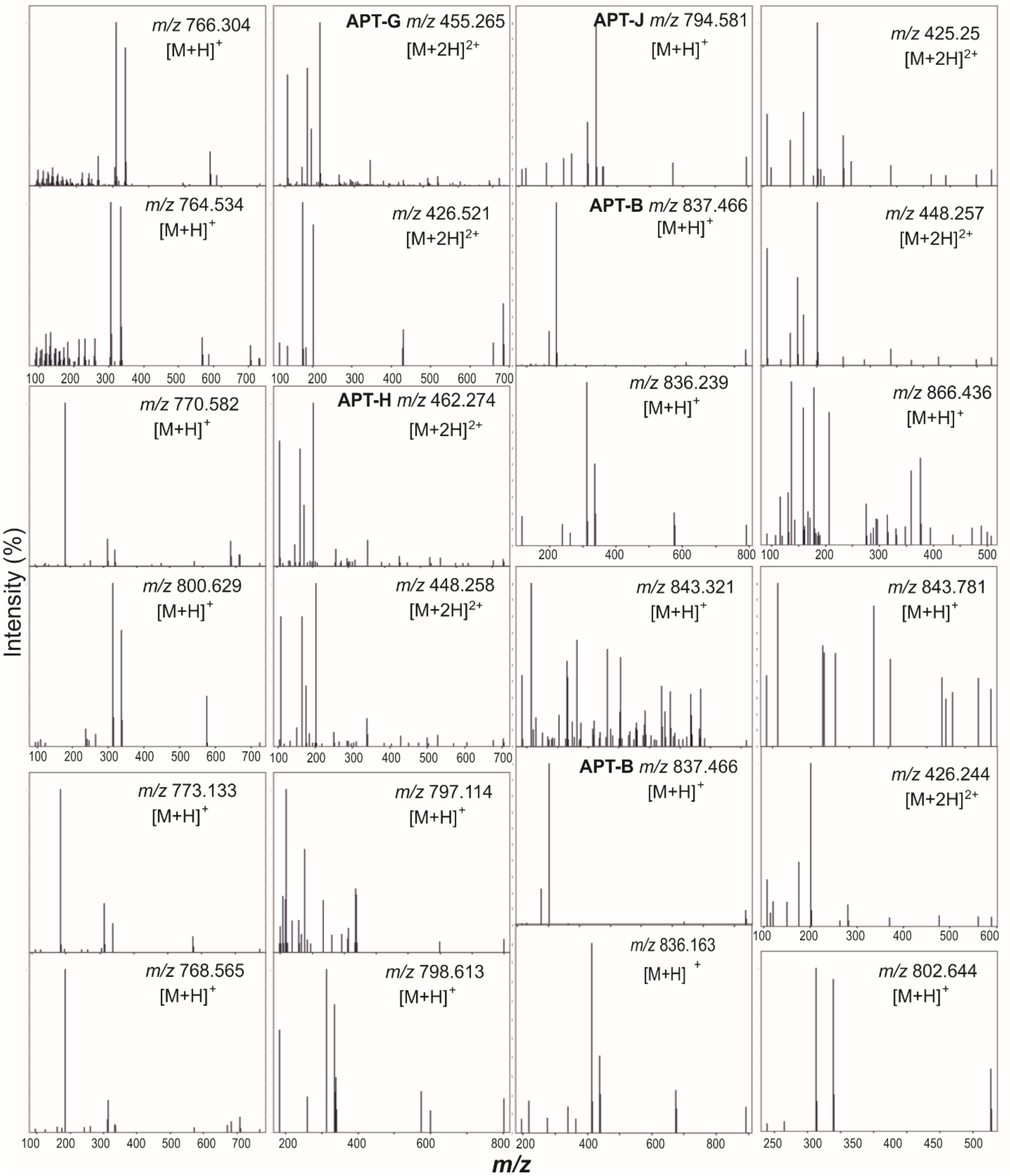
m/z 766.304 [M+H]+
APT-G m/z 455.265 [M+2H]2+
APT-J m/z 794.581 [M+H]+
m/z 425.25 [M+2H]2+
m/z 764.534 [M+H]+
m/z 426.521 [M+2H]2+
APT-B m/z 837.466 [M+H]+
m/z 448.257 [M+2H]2+
m/z 836.239 [M+H]+
m/z 866.436 [M+H]+
m/z 770.582 [M+H]+
APT-H m/z 462.274 [M+2H]2+
m/z 800.629 [M+H]+
m/z 448.258 [M+2H]2+
m/z 843.321 [M+H]+
m/z 843.781 [M+H]+
APT-B m/z 837.466 [M+H]+
m/z 426.244 [M+2H]2+
m/z 773.133 [M+H]+
m/z 797.114 [M+H]+
m/z 836.163 [M+H]+
m/z 802.644 [M+H]+
m/z 768.565 [M+H]+
m/z 798.613 [M+H]+
Intensity (%)
m/z

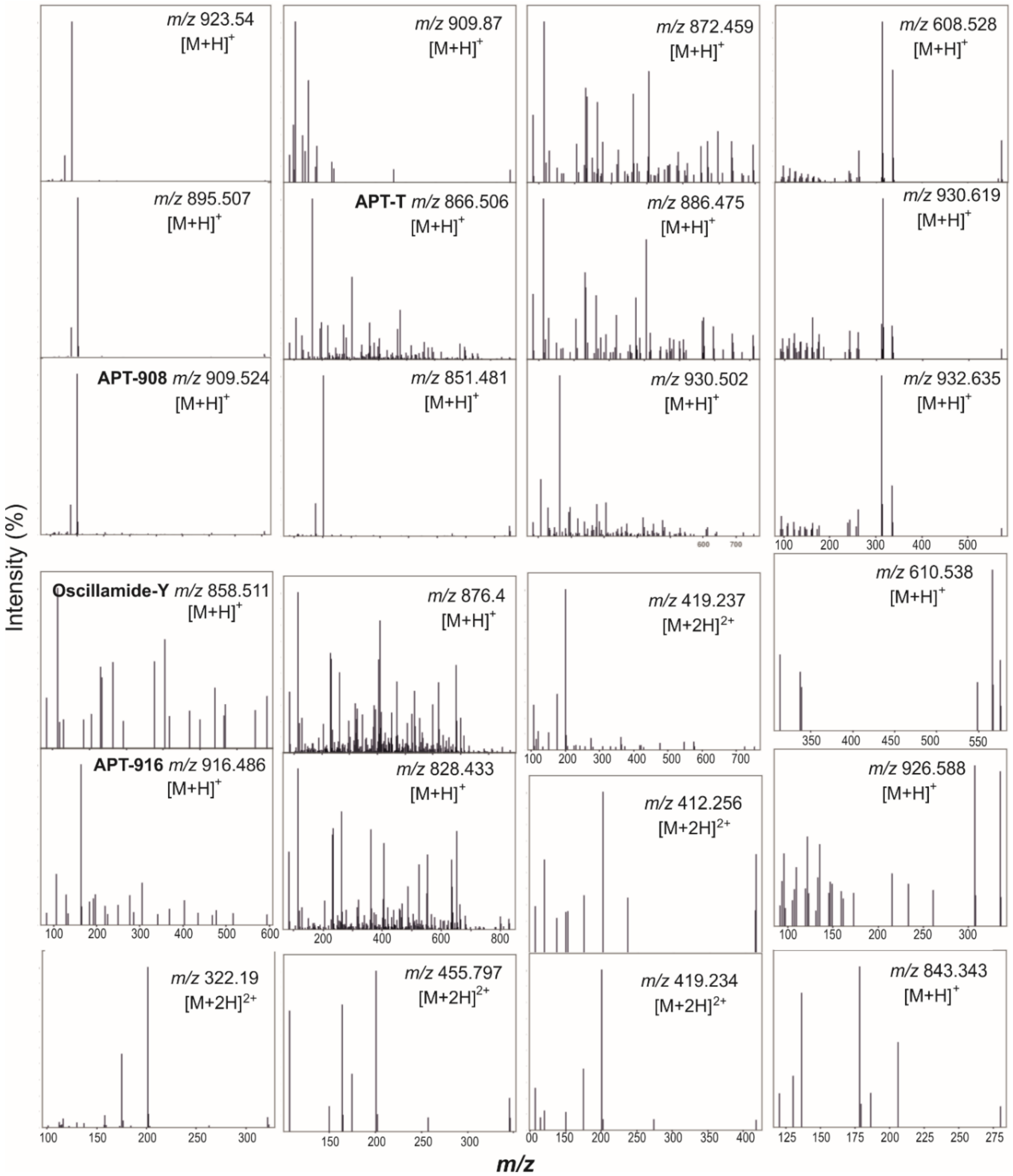

Figure S3. HR-MS/MS product ion spectra of protonated known/unknown APTs forming five clusters (Figure 2).

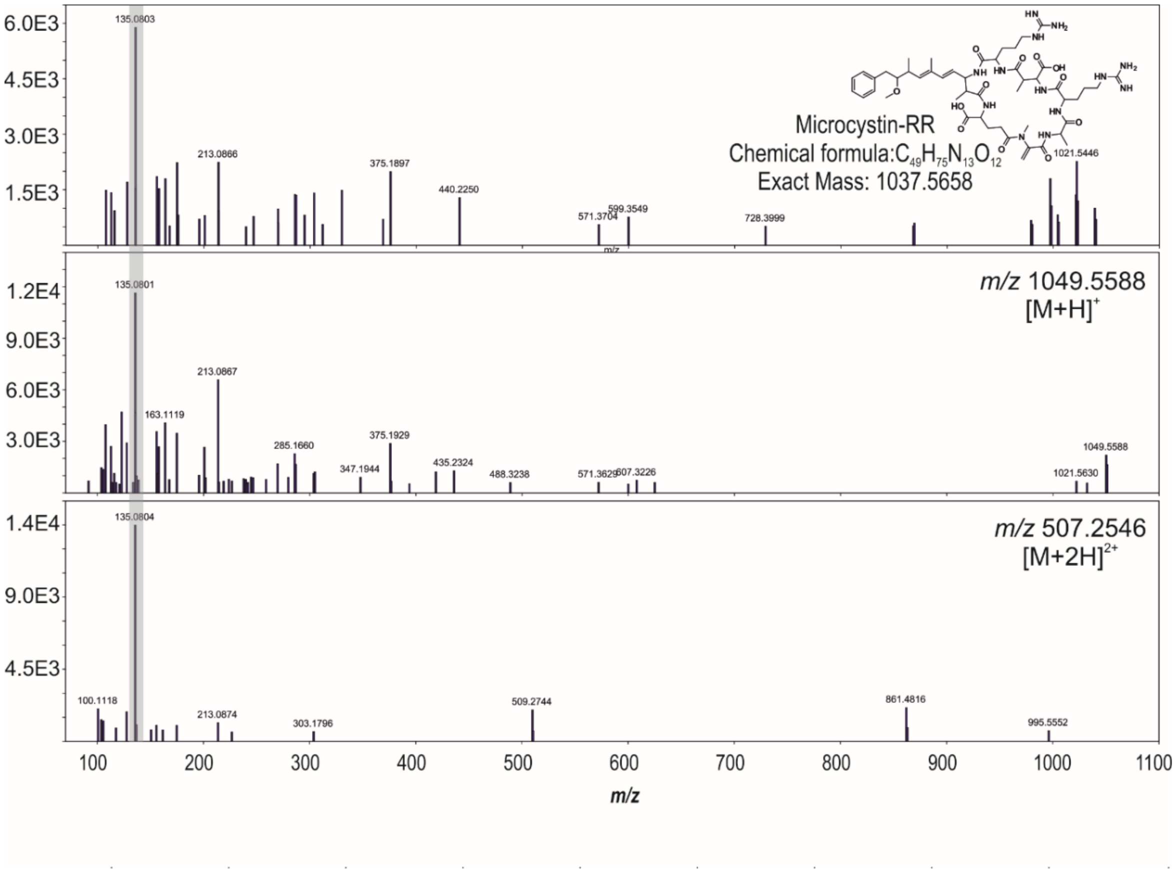

Figure S4. HR-MS/MS product ion spectra MC-RR with comparison with two unknown variants highlighting the presence of diagnostic ion peak originating from Adda moiety at m/z 135.0804 Da.

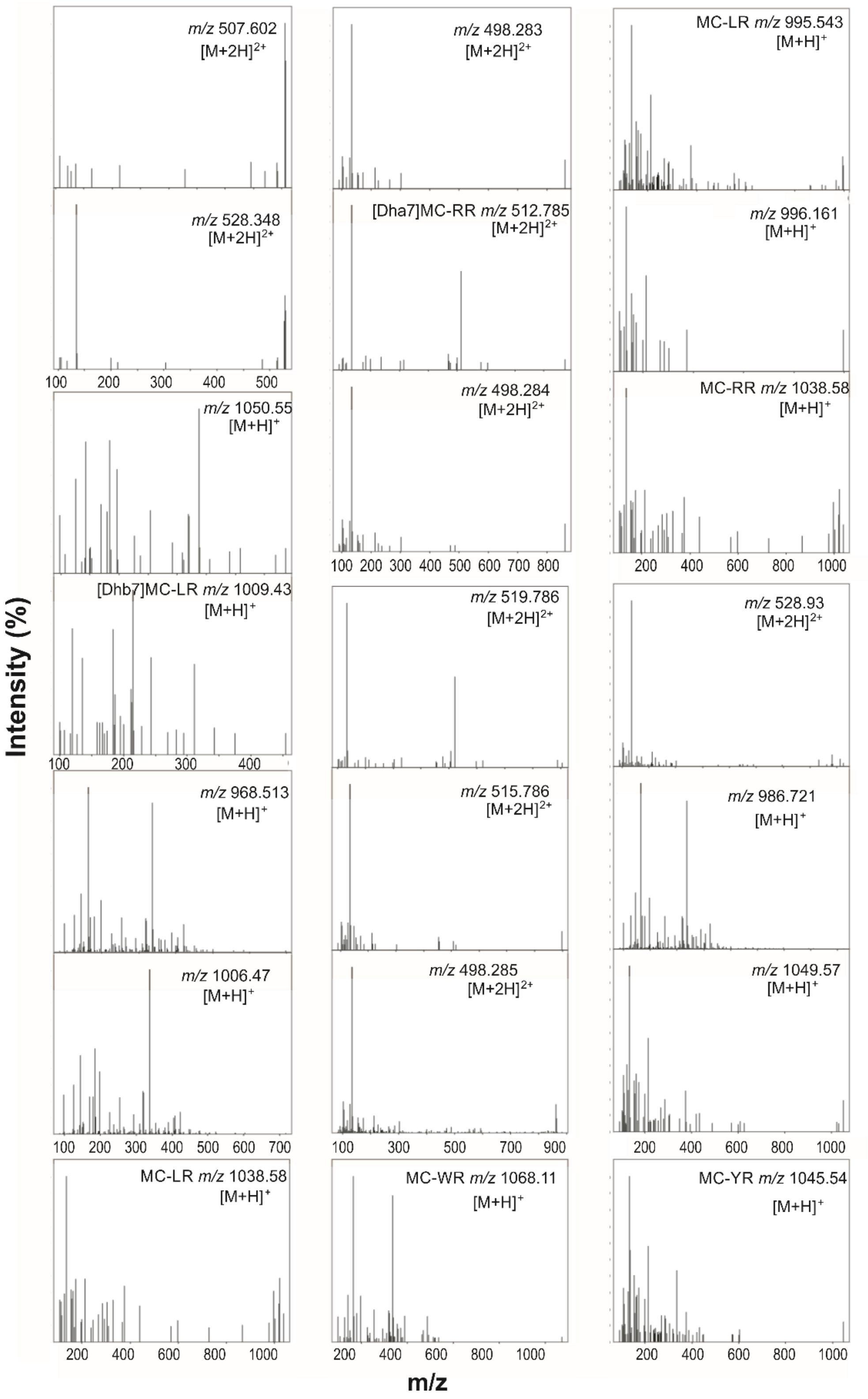

Figure S5. HR-MS/MS product ion spectra of protonated known/unknown MCs forming two clusters (Figure 2).

Code 1: The R code developed for the entire analysis.

```
# Install/Load libraries
install.packages("ISLR")
install.packages("heatmaply")
library(ISLR)
library(ggplot2)
library(reshape2)
library(car)
library(heatmaply)

# Import data
cyanos <- read.table("cyanos.txt", header = T, sep = '\t', row.names = 1)
pept <- read.table("peptides.txt", header = T, sep = '\t', row.names = 1)

# Transpose data - the regression function is made for colums
cyanos_t1 <- t(cyanos)
pept_t <- as.data.frame(t(pept))

d <- as.data.frame(cbind(cyanos_t1, row.names(cyanos_t1)))
m_cya = melt(d, id.vars="V30")
m_cya$V30 = factor(m_cya$V30,
          levels=c("KL.Apr",    "KL.May",    "KL.Jun",    "KL.Jul","KL.Aug",
              "KL.Sep",  "DH.Apr",    "DH.May",    "DH.Jun",    "DH.Jul",
              "DH.Aug",  "DH.Sep",    "KV.Apr",    "KV.May",    "KV.Jun",
              "KV.Jul",  "KV.Aug",    "KV.Sep"))
p <- ggplot(data=m_cya, aes(x=V30, y=as.numeric(value), fill=V30)) +
 geom_bar(stat="identity") +
 scale_fill_viridis_d() +
```

```
 facet_wrap(~ variable, scales = "free", ncol=6) +
 scale_y_continuous(name="Abundance") +
 theme_minimal()

p + theme(legend.position = "none",
   axis.text.x = element_text(angle = 90),
   strip.text = element_text(face = "italic"))

# Explore and plot the cyano data
summary(cyanos_t1)

# Logistics Regression
res_pval <- matrix(NA, nrow = ncol(cyanos_t1), ncol = ncol(pept_t))
row.names(res_pval) <- colnames(cyanos_t1)
colnames(res_pval) <- colnames(pept_t)


for(i in 1:ncol(cyanos_t1)){
 for(j in 1:ncol(pept_t)){
   glm.fit <- glm(pept_t[,j] ~ as.numeric(cyanos_t1[,i]), family = binomial())
   fit <- Anova(glm.fit, type = 2)
   res_pval[i,j] <- fit$`Pr(>Chisq)`  #  gives the p-value for an asymptotic chi square statstic based on the deviance
 }
}

# Plot the results as heatmap
# Remove unsignificant values
filt_res <- matrix(NA, nrow = ncol(cyanos_t1), ncol = ncol(pept_t))
```

```
row.names(filt_res) <- colnames(cyanos_t1)

colnames(filt_res) <- colnames(pept_t)

for(i in 1:nrow(res_pval)){

  for(j in 1:ncol(res_pval)){

    if(res_pval[i,j]>=0.05){

      filt_res[i,j] <- NA

    }

    else{

      filt_res[i,j] <- res_pval[i,j]

    }

}}


# Make the species name italic

rownames(filt_res) <- paste("<i>", rownames(filt_res), "</i>")

# Plot raw p-values

heatmaply(filt_res, na.omit = T, na.value = "grey50", Rowv = F, Colv = F,

          grid_color = "grey50", key.title = "P - value", xlab = "Cyanopeptides",

          ylab = "Species")

# Save these results to a file

write.table(filt_res, "Logistic-regression_Cyanos_raw_Pval.txt", sep = '\t')


# Adjust these p-values using the Benjamini-Hochberg procedure

fdr_res <- matrix(NA, nrow = ncol(cyanos_t1), ncol = 1)

row.names(fdr_res) <- colnames(cyanos_t1)


for (i in 1:ncol(res_pval)){

  df <- res_pval[order(res_pval[,i]), i]

  v <- 1 ; s <- 1

  p_fdr <- c()
```

```
  for(i in 2:length(df)){
    if(df[i]==df[i-1]){
      v <- c(v, s)
    }
    else{
      s = s+1
      v <- c(v, s)
    }
  }
  df <- cbind(df, v)
  f <- df[row.names(res_pval),]  # Reorder them as in the initial table (res_pval)
  for(i in 1:nrow(f)){
    fdr <- f[i,1]*(nrow(f)/f[i,2])
    if(fdr > 1){
      p_fdr <- c(p_fdr, 1)
    }
    else{
      p_fdr <- c(p_fdr, fdr)
    }
  }
  #df <- cbind(df, p_fdr)
  fdr_res <- cbind(fdr_res, p_fdr)
}


fdr_res <- fdr_res[,-1]
colnames(fdr_res) <- colnames(res_pval)




# Remove unsignificant values
```

```
filt_res_fdr <- matrix(NA, nrow = ncol(cyanos_t1), ncol = ncol(fdr_res))

row.names(filt_res_fdr) <- colnames(cyanos_t1)

colnames(filt_res_fdr) <- colnames(fdr_res)

for(i in 1:nrow(fdr_res)){

  for(j in 1:ncol(fdr_res)){

    if(fdr_res[i,j]>=0.2){

      filt_res_fdr[i,j] <- NA

    }

    else{

      filt_res_fdr[i,j] <- fdr_res[i,j]

    }

  }}


# Make the species name italic

rownames(filt_res_fdr) <- paste("<i>", rownames(filt_res), "</i>")

# Plot raw p-values

heatmaply(filt_res_fdr, na.omit = T, na.value = "grey50", Rowv = F, Colv = F,

          grid_color = "grey50", key.title = "P - value", xlab = "Cyanopeptides",

          ylab = "Species")

# Save these results to a file

write.table(filt_res_fdr, "Logistic-regression_Cyanos_FDR_Pval.txt", sep = '\t')
```
